# Supplementary material for: Characterization of De Novo Promoter Variants in Autism Spectrum Disorder with Massively Parallel Reporter Assays
Source: Int J Mol Sci. 2023 Feb 9;24(4):3509. doi: 10.3390/ijms24043509 (PMC9959321; doi:10.3390/ijms24043509)
Supplement: Supplementary file 1 [file ijms-24-03509-s001.zip › ijms-2060290-supplementary/ijms-2060290-supplementary.pdf]

Autism MPRA Manuscript supplementary

Supplementary Note 1

Comparison between cases and control does not exhibit significant differences

As stated in the main text, we were unable to find significant differences between the HcDNV cases and controls. We first tested for differences of enrichments in predictive annotation categories used by An et al. 2018 (*7*) and found that none of the annotations had significantly more cases or controls. (**Methods; Supplementary Figure 5a**). We next compared the number of variants that are found overlapping ATACseq peaks and H3K27ac ChIPseq peaks for ESC and NPC cell types (**Methods**) and find no significant differences between the number of case variants and control variants found in either ATACseq or H3K27ac peaks (ATACseq peaks: Fisher exact test p-value 0.1385, odds ratio 2.8676. H3K27ac Peaks: fisher exact test p-value 0.4724, odds ratio NA). Additionally, we are unable to see differences in the number of case or control variants found in ESC peaks compared to NPC peaks (*19*) (ATACseq peaks: Fisher exact test p-value 0.6312, odds ratio 0.8329. H3K27ac Peaks: Fisher exact test p-value 0.3523, odds ratio 0.7443) (**Methods; Supplementary Figure 5b**).

We next utilized the nearest gene in genomic coordinates to the variant to observe any gene level differences. Since the variants are promoter based they are likely to affect the closest gene. To find if these variants are disrupting gene expression by impacting transcription factor binding sites, we utilized the program fimo ('Find Individual Motif Occurrences') ((*33*); **Methods**) which scans a set sequences for individual matches to each of the motifs provided by the user. We find that there is no significant difference in the number of transcription factor binding motifs interacting with the variant location with the reference allele (Fisher exact test p-value: 0.2842, odds ratio: 0.8252). Additionally, when looking for differences in motif disruption, we observe no significant differences between cases and controls for motifs gained or lost when mutating from the reference allele to the alternate allele (Fisher exact test p-value 0.5882, odds ratio 0.8805).

Next, we wanted to examine if there is a difference in the genes that are downstream of these variants to see if they are involved in different pathways. We took the associated genes (as described above) and tested them with Gene Set Enrichment Analysis (GSEA) (*34*) to find processes that are enriched with our gene set (**Methods**). In addition we utilized the program DOMINO which uses databases of interaction to find subnetworks of proteins within our gene set that are empirically enriched for cellular processes (*34*) (**Methods**). In both instances we do not observe differences in the top biological processes enriched with the case or control variants or the number and processes of interacting proteins. Finally, to examine if these variants are involved in an intermediate chain in ASD networking, we added previously disease associated ASD and DDD genes reported by Fu et al. 2021 (*24*) to the genes affected by the variants. The GSEA and DOMINO results show strong enrichments, but it appears that the enrichment and the interaction plots are mostly driven by the ~800 ASD and DDD genes with the HcDNV cases or HcDNV controls having little involvement. We then tested if either the variants' nearby gene or a transcription factor that interacted with the variant was enriched for a gene in this ASD and DD gene list, and found no significant differences (Fisher exact test p-value: 0.7349, odds ratio: 0.8745). We repeated the above analysis comparing HcDNV inhibitor cases to HcDNV inhibitor controls as well as only activator DNVs, but were unable to find any significant differences between them for any of the above mentioned analyses.

Supplementary Note 2

No significant interaction between HcDNVs and previously reported ASD genes

We wanted to test if our HcDNVs were involved in ASD at an intermediate stage of the mechanistic process. To accomplish this, we utilized a list of ~800 ASD and DDD associated genes reported by Fu et al. (*24*). Using the program fimo, we find that the transcription factors that are having their binding motifs lost or gained by the variant allele of HcDNVs are not enriched for ASD associated genes (Fisher exact test p-value: 0.9072; odds ratio: 0.9677). Additionally, we found no significance in HcDNVs having their nearest gene be in this list compared to the number of background variants (Fisher exact test p-value: 0.247) but note that 3 high confident case variants are nearest the ASD associated genes: KCNB1, SATB1, and AGO4. And when looking at protein interaction and enrichment of cellular processes, we found that the ASD genes were driving all the significance and removing the HcDNV variants did not change the level of significance in these interactions.

Supplementary Figure S1

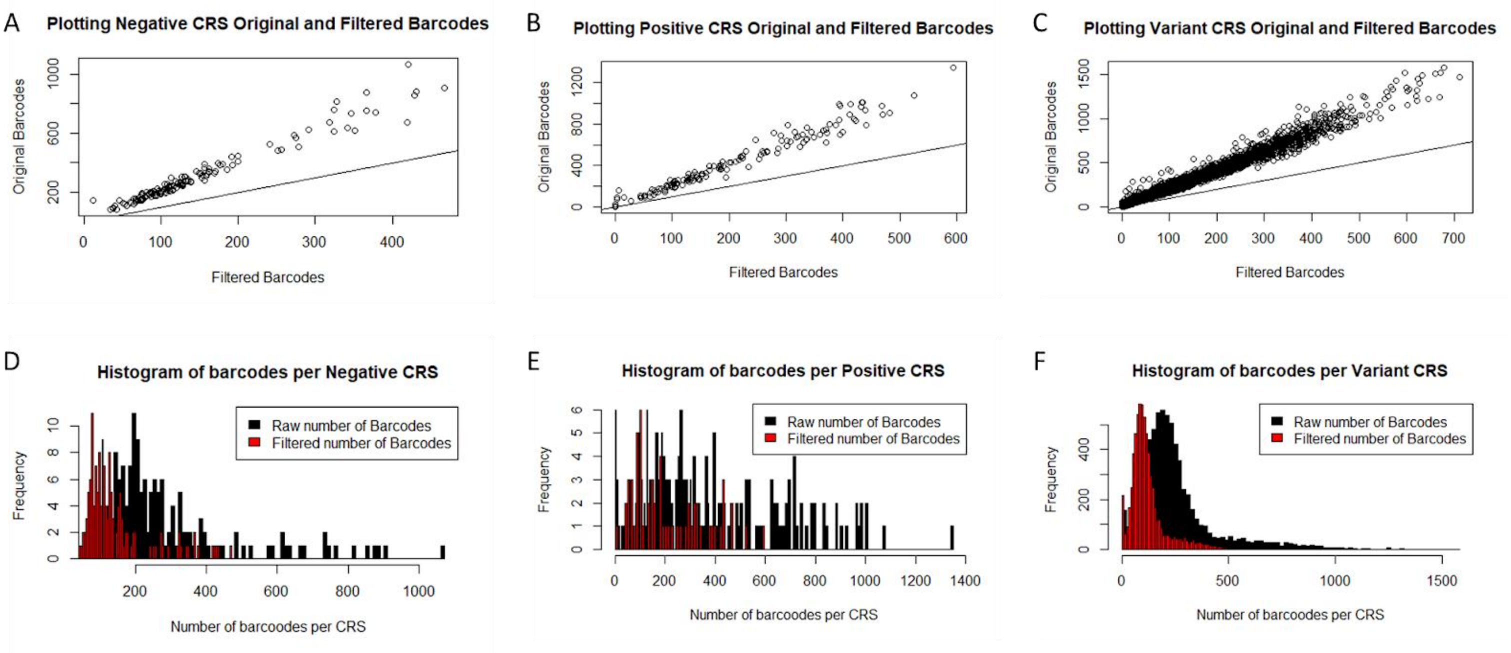

Figure S1. MPRAflow Association filtering of barcodes.

A,B,C) Scatterplot of the number of barcodes before and after filtering for each CRS. Black line denotes diagonal of no change between original and filtered number of barcodes A) Plot for negative control CRSs B) Plot for positive control CRSs C) Plot for all reference and alternate CRSs **D,E,F)** Histogram of the barcodes before and after filtering each CRS. D) Plot for negative control CRSs **E)** Plot for positive control CRSs F) Plot for all reference and alternate CRSs

Supplementary Figure S2

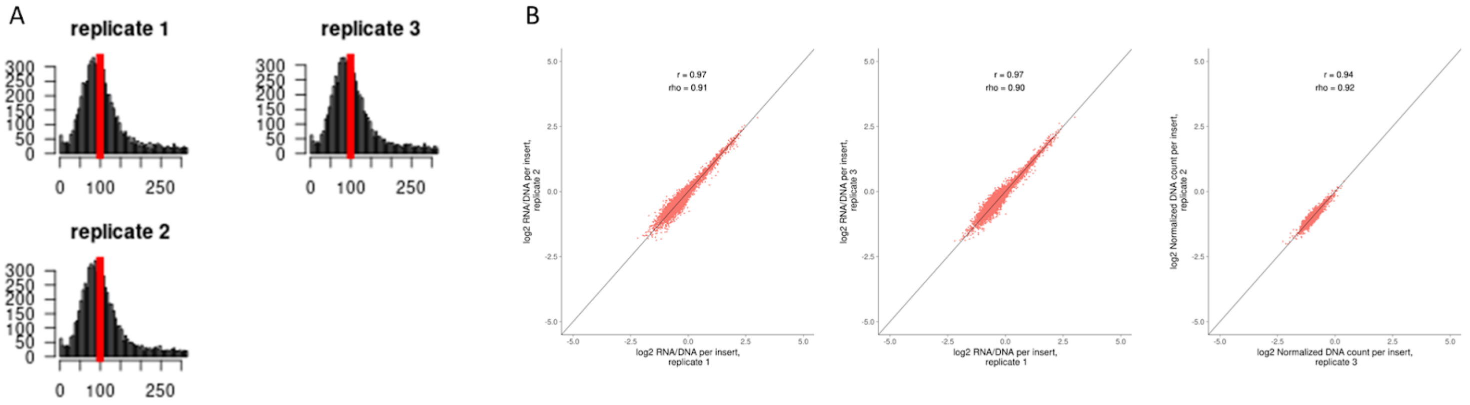

Figure S2. MPRAflow counting of barcodes.

A) Histogram of the number of barcode for each replicate after filtering with the count command. **B)** Scatterplot of the ratio of RNA counts to DNA counts between the 3 different replicates.

Supplementary Figure S3

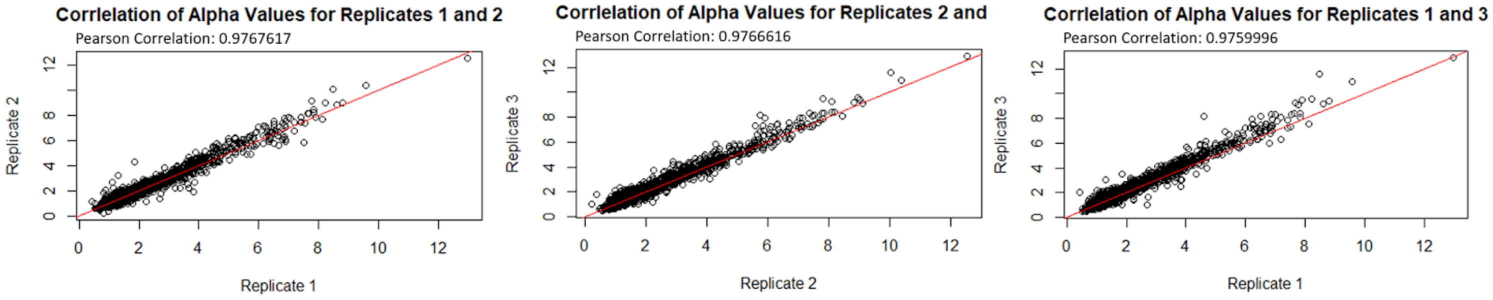

Figure S3. MPRAnalyze Alpha value correlation between replicates.

Scatterplot comparing the transcription rate “alpha value” reported by MPRAnalyze between the 3 replicates.

Supplementary Figure S4

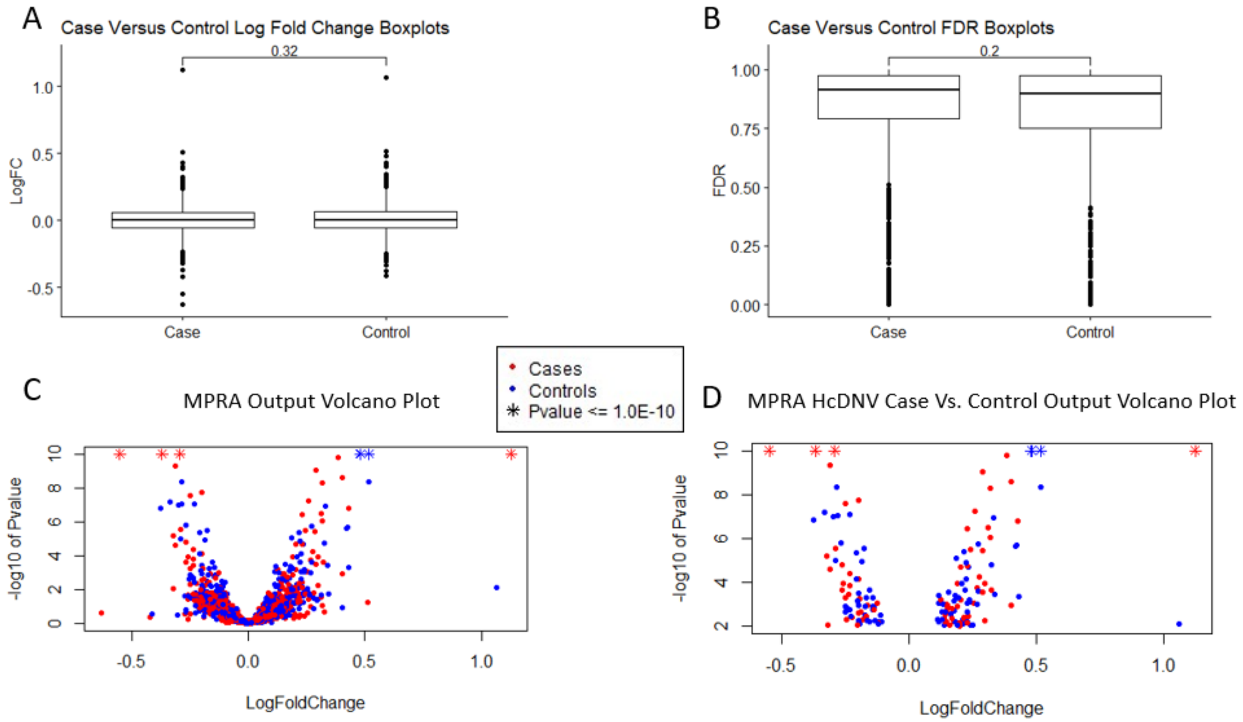

Figure S4. Comparison of case versus control MPRAnalyze output.

A) Boxplot comparing the log fold change between all cases and controls. **B)** Boxplot comparing FDR values for all cases and controls. **C,D)** Volcano plot showing the natural log fold change between alternate allele alpha value over reference allele alpha value on the x-axis and the –log10 of the MPRAnalyze p-value reported for the differential expression on the y-axis. **C)** Volcano plot comparing all cases and controls. **D)** Volcano plot comparing only the HcDNV cases and controls.

Supplementary Figure S5

A

Name	Pvalue	Odds Ratio	Name	Pvalue	Odds Ratio
PriorFindingsIn.Family	0.750062	0.730094	C63	0.787353	1.306286
ActiveTSS	1	0.894118	CHD8.target	0.359893	0.595933
Conserved.Loci	0.242423	1.553281	PhastCons	0.314133	1.469816
Lasso	0.423113	2.302128	PhyloP	1	0.990468
DAWN	1	NA	ChmE1	0.403987	0.72004
C7	0.230952	2.371369	ChmE2	0.86333	1.097638
C12	0.314133	1.469816	ENCODE_TFBS	1	1.010878
C18	0.667979	0.588235	REP_DNase	0.725984	0.861567
C20	1	0.990468	ENCODE_DNase	0.258599	0.576921
C26	0.603066	2.267241	MidFetal_H3K27ac	1	1.038923
C28	0.359893	0.595933	ASD_TADA_FDR01	1	NA
C42	0.603066	2.267241	ASD_TADA_FDR03	1.00E+00	0
C49	1	1.12069	PLI_over90	0.265321	0.646381
			DevDelay	0.603066	2.267241

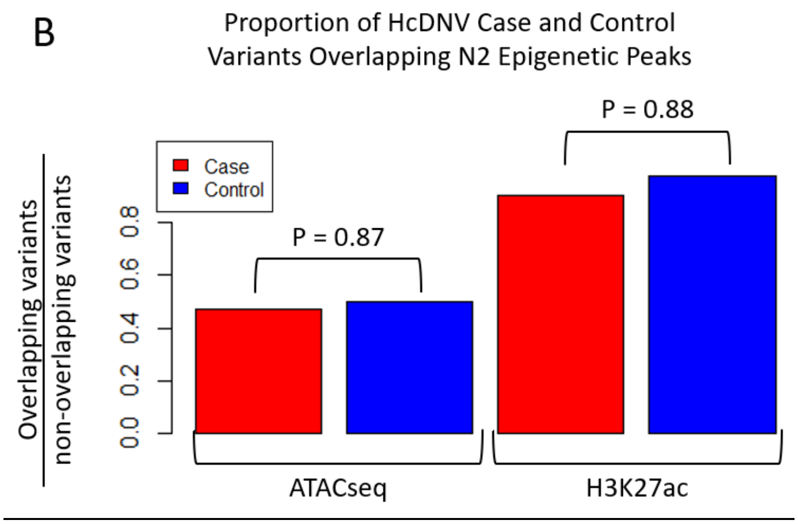

Figure S5. Analysis of HcDNV cases and control variants.

A) Table of fisher exact test p-value and odds ratio for the 28 annotation categories. **B)** Barplot comparing the proportion of variants overlapping ATAC-seq and H3K27ac ChIP-seq epigenetic peaks to the variants that do not overlap the epigenetic peaks.

Supplementary Figure S6

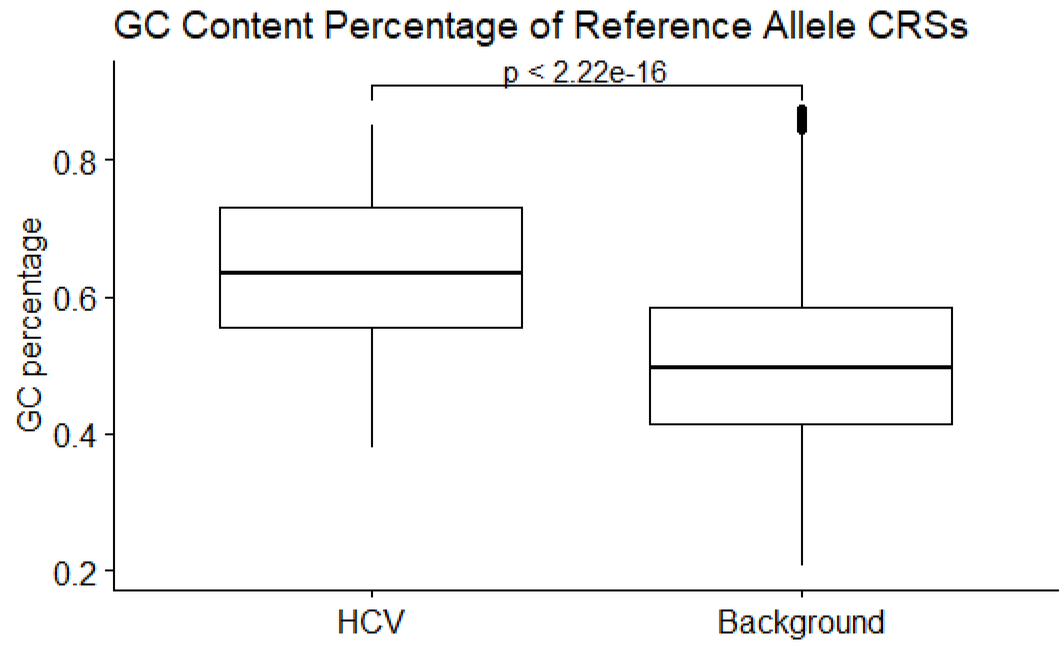

Figure S6. Comparing GC content between HcDNVs and Background variants.

A) Boxplot of the GC content percentage for HcDNV variants and the Background variants. Wilcoxon rank sum test p-value: $3.92*10^{-36}$

Supplementary Table S1

	Starting	Association	Count	MPRAnalyze Input
**Positive**	150	147	144	
**Negative**	150	149	149	
**Variants**	7200	6708	6589	6180 (3090 pairs)
**Filters:**	NA	Cigar=200M	Barcode length= 15	
**Filters:**	NA	Min-frac=0.7	Merge-intersect=true	
**Filters:**	NA	Mapq=1	Mpranalyze=true	

Supplementary Table S2

	Cases	Controls	Activators	Inhibitors
HcDNV	78	87	96	69
Background	1554	1371	1487	1438

	HcDNV Activator	HcDNV Inhibitor	Background Activator	Background Inhibitor
Case	48	30	770	784
Control	48	39	717	654

Supplementary Table S5

Name	Sequence
5BC-AG-f01	CTCACTCAGCCTGCATTTCTGCCAGGGCCCGCTCTAGACCTG CAGGAGGACCGGATCAACT
5BC-AG-r01	GCTTTCGCTTAGCGATGTGTTCACTTTGCACAGTACCGGATTG CCAAGCTGGAAGTCGAGCTTCCATTATATACCCTCTAGTGTCG GTTCACGCAATG
5BC-AG-f02	CTCACTCAGCCTGCATTTCTG
5BC-AG-r02	TGAACAGCTCCTCGCCCTTGCTCACCATGGTGGCGACCGGTN NNNNNNNNNNNNNNCTTAGCTTTCGCTTAGCGATGTGTTC
P7-pLSmp-ass-gfp	CAAGCAGAAGACGGCATACGAGATGCTCCTCGCCCTTGCTCA CCATG
P5-pLSmP-ass-i#	AATGATACGGCGACCACCGAGATCTACAC##########CAGCC TGCATTTCTGCCAGGG (########## represents index sequence for multiplexing)
P7	CAAGCAGAAGACGGCATACGAGAT
P5	AATGATACGGCGACCACCGAGATCTACAC
P7-pLSmp-assUMI-gfp	CAAGCAGAAGACGGCATACGAGATNNNNNNNNNNGCTCCTCG CCCTTGCTCACCATG
P5-pLSmP-5bc-i#	AATGATACGGCGACCACCGAGATCTACAC##########GCAAA GTGAACACATCGCTAAGCGAAAGC (########## represents index sequence for multiplexing)
pLSmP-ass-seq-R1	GGCCCGCTCTAGACCTGCAGGAGGACCGGATCAACT
pLSmP-ass-seq-R2	CATTATATACCCTCTAGTGTCGGTTCACGCAATG
pLSmP-ass-seq-ind1	GCAAAGTGAACACATCGCTAAGCGAAAGCTAAG
pLSmP-rand-ind2	TCTAGAGCGGGCCCTGGCAGAAATGCAGGCTG
pLSmP-bc-seq	GCTCCTCGCCCTTGCTCACCATGGTGGCGACCGGT
pLSmP-UMI-seq	ACCGGTCGCCACCATGGTGAGCAAGGGCGAGGAGC
pLSmP-5bc-seq-R2	CTTAGCTTTCGCTTAGCGATGTGTTCACTTTGC
